# Performance of Multiplex Cytokine Assays in Serum and Saliva among Community-Dwelling Postmenopausal Women

**DOI:** 10.1371/journal.pone.0059498

**Published:** 2013-04-05

**Authors:** Richard W. Browne, Alpdogan Kantarci, Michael J. LaMonte, Christopher A. Andrews, Kathleen M. Hovey, Karen L. Falkner, Ali Cekici, Danielle Stephens, Robert J. Genco, Frank A. Scannapieco, Thomas E. Van Dyke, Jean Wactawski-Wende

**Affiliations:** 1 Department of Biotechnical and Clinical Laboratory Sciences, University at Buffalo, Buffalo, New York, United States of America; 2 Department of Periodontology, The Forsyth Institute, Boston, Massachusetts, United States of America; 3 Department of Social and Preventive Medicine, University at Buffalo, Buffalo, New York, United States of America; 4 Department of Biostatistics, University at Buffalo, Buffalo, New York, United States of America; 5 Department of Oral Biology, University at Buffalo, Buffalo, New York, United States of America; University of Toronto, Canada

## Abstract

Multiplexing arrays increase the throughput and decrease sample requirements for studies employing multiple biomarkers. The goal of this project was to examine the performance of Multiplex arrays for measuring multiple protein biomarkers in saliva and serum. Specimens from the OsteoPerio ancillary study of the Women’s Health Initiative Observational Study were used. Participants required the presence of at least 6 teeth and were excluded based on active cancer and certain bone issues but were not selected on any specific condition. Quality control (QC) samples were created from pooled serum and saliva. Twenty protein markers were measured on five multiplexing array panels. Sample pretreatment conditions were optimized for each panel. Recovery, lower limit of quantification (LLOQ) and imprecision were determined for each analyte. Statistical adjustment at the plate level was used to reduce imprecision estimates and increase the number of usable observations. Sample pre-treatment improved recovery estimates for many analytes. The LLOQ for each analyte agreed with manufacturer specifications except for MMP-1 and MMP-2 which were significantly higher than reported. Following batch adjustment, 17 of 20 biomarkers in serum and 9 of 20 biomarkers in saliva demonstrated acceptable precision, defined as <20% coefficient of variation (<25% at LLOQ). The percentage of cohort samples having levels within the reportable range for each analyte varied from 10% to 100%. The ratio of levels in saliva to serum varied from 1∶100 to 28∶1. Correlations between saliva and serum were of moderate positive magnitude and significant for CRP, MMP-2, insulin, adiponectin, GM-CSF and IL-5. Multiplex arrays exhibit high levels of analytical imprecision, particularly at the batch level. Careful sample pre-treatment can enhance recovery and reduce imprecision. Following statistical adjustments to reduce batch effects, we identified biomarkers that are of acceptable quality in serum and to a lesser degree in saliva using Multiplex arrays.

## Introduction

Accurate and reliable measurement of inflammatory biomarkers is critical to assessing inflammatory mechanisms involved in many diseases including periodontal disease. Periodontitis is a good model for studying these biomarker issues because although the etiology of periodontitis is bacterial, the pathogenesis is clearly inflammatory [1]. Inflammation is a complex process that involves multiple key mediators [2] including chemokines, pro- and anti-inflammatory cytokines, growth factors, angiogenesis factors, and protein hormones. In order to thoroughly evaluate the etiological role of inflammatory processes in the oral and systemic compartments, it is necessary to quantify concentrations of relevant biomarkers in fluids such as serum, gingival crevicular fluid, and saliva. Given its ease of collection and growing appreciated relevance to physiological and pathological events in the human body, there is recent interest in the use of saliva as a diagnostic biological fluid to potentially discriminate oral and systemic pathologies from health. Saliva presents specific measurement challenges due to its viscosity, differences in matrix, and molecular content. It is also not known how comparable the content of saliva is to the widely used serum in screening for biological changes indicative of disease onset or progression. High-throughput measures of analytes in saliva and serum therefore offer a novel and convenient method for comparing and assessing the role of biomarkers in oral and systemic compartments. These methods need to be efficient with respect to cost and sample volume requirements while also being accurate and reproducible in characterizing “health” and “disease.”

Multiplex array platforms and associated reagent kits have been developed which assay for a large number of analytes and have the ability to rapidly process multiple specimens. These systems are more cost-effective and increase the throughput and decrease the sample amounts compared with traditional EIA and ELISA. With applications ranging from protein to nucleic acids multiplex assays add value in their ability to screen multiple biomarkers where there is no know correlate or identify complex and dynamic biosignatures that offer better differentiation than any single biomarker can afford. Bead-based flow cytometric multiplex arrays are commonly used and commercially available for the detection of proteins. The technique utilizes microsphere beads, coated with monoclonal antibodies against specific proteins, to measure analyte concentrations in body fluids, cell extracts and culture supernatants [3]–[5]. Data acquired through multiplex arrays have compared similarly to measures from conventional techniques such as enzyme linked immunosorbent assay (ELISA) [6], [7]. The cost/benefit ratio of this technology has also been reportedly favorable to conventional bioassay methods in terms of time, labor, cost, and particularly sample volume. Typically, 5–25 µl of sample is sufficient for multiple target detection which offers considerable advantage when limited research study samples of serum, plasma or bodily fluid may be available. In addition, simultaneous assessment of multiple analytes by multiplex techniques avoids the need for diluting samples multiple times or for multiple freeze-thawing of samples, each of which can affect measurement accuracy and precision.

A large number of studies have reported inflammatory biomarker concentrations in samples tested using multiplex arrays with little apparent attention in the manuscripts to quality control (QC) performance. Few reports have been published on methodological limitations and imprecision estimates of this technique in blood serum and plasma [8]–[10] and information for saliva and other medium is even more sparse. This issue becomes even more critical when examining and comparing analyte concentrations in different biological fluids with distinct matrix characteristics that impact assay performance. Therefore, there is a need for independent systematic characterization of the assays and their performance, in saliva as well as in serum. Our goal in the present study was to use multiplex techniques and vendor supplied assay panels to measure a defined set of protein biomarkers in homologous serum and saliva samples, choose dilutions and diluents to enhance assay performance, and examine the performance characteristics of these assays. In this report we describe the ability of these assays to provide reliable measurements of inflammatory cytokines and other biomarkers in homologous samples of serum and saliva collected from participants in the Buffalo OsteoPerio Study, ancillary to the national Women’s Health Initiative Observational Study.

## Materials and Methods

### Participants

This investigation is part of a larger study funded under the ARRA program by NIDCR to characterize biomarkers of inflammation in both serum and saliva, determine the extent that serum and saliva measures correlate, and to determine associations of serum and salivary markers with clinical periodontal disease and bone density measures in an established and well-characterized cohort of postmenopausal women. Serum and saliva samples were previously collected as part of two completed studies on osteoporosis and periodontal disease (OsteoPerio Studies) that were ancillary to the Buffalo center of the national Women’s Health Initiative Observational Study (WHI-OS). Participants for the OsteoPerio studies were recruited from 2,249 postmenopausal women ages 53–84 years who enrolled in the WHI-OS at the University at Buffalo clinical center of the WHI. The baseline OsteoPerio study enrolled 1,362 women and 1,025 of these women were reexamined five years later through a second examination. The OsteoPerio baseline visit corresponded with the 3^rd^ annual visit of the WHI-OS. The OsteoPerio studies included questionnaires on demographics, lifestyle, and medical history; dual energy x-ray absorptiometry (DXA) scans for measuring bone density; and a comprehensive clinical dental exam with oral radiographs [11] [12]; [13]. In addition, all the information from the parent WHI-OS was available for use in the OsteoPerio studies. Inclusion criteria included having six intact teeth and no major disease diagnoses. All women provided written informed consent and all studies have been approved by the Health Sciences Institutional Review Board at the University at Buffalo.

### Specimens

Collection of serum and saliva was completed as part of the OsteoPerio studies. In brief, participants came to the Buffalo WHI clinic in the morning and provided a fasting saliva and blood sample. All samples were collected, processed and stored using standardized protocols.

Saliva samples were collected in the clinic prior to blood draw, eating/drinking or dental examination. Participants provided 5 ml of saliva in a pre-marked collection tube. Saliva collection was completed in 10 minutes or less. Those with difficulty producing enough saliva were offered the option to chew a sterile rubber band to help stimulate saliva production. Samples were transferred into 0.5 ml cryogenic storage straws, which were sealed and placed in −80°C freezers for 24 hours prior to long-term submersion in liquid nitrogen (−196°C).

Fasting blood samples were collected at the same visit by venipuncture after the saliva collection and prior to the dental examination. A 10cc tube without anticoagulant was used for serum collection. The tube was placed in darkness for 30 minutes to allow a clot to form and centrifuged at 1500×g for 15 minutes. The serum portion was removed, transferred to 0.5 ml straws, sealed and placed in −80°C freezers for 24 hours prior to long-term submersion in liquid nitrogen (−196°C).

For the purpose of this study, quality control (QC) specimens were created from serum and saliva samples obtained at a single visit from 24 individual volunteers, using a protocol identical to that used for participant samples. Samples were centrifuged and pooled into a single sterile flask. The pooled specimens were then centrifuged again and portioned into 0.5 ml cryogenic storage straws (125 serum and 125 saliva straws), heat sealed and placed stored in liquid nitrogen (−196°C).

For analysis, cryogenic straws of all samples were retrieved from liquid nitrogen, placed on dry ice and shipped to a single research laboratory facility (The Forsyth Institute, Boston, MA). They remained in −80°C freezers until the time of testing.

Samples were sent in batches that included at least two serum and saliva QC samples. The samples were assembled and sent in blinded fashion as related to health outcomes and any personal information. The samples from one individual who had two time points were sent in a single batch to be assayed on the same plate. The order of samples on the plate was pre-determined for the laboratory to follow. All samples were blinded to the laboratory personnel by use of unique sample identification numbers. The present study includes stored serum samples from 910 women at baseline and from 410 women at follow-up, among these were 1133 paired saliva/serum samples (725 baseline and 408 follow-up pairs).

### Multiplex Cytokine and Inflammatory Biomarker Analysis

Multiplexed sandwich immunoassays, based on flowmetric Luminex™ xMAP technology, were conducted at The Forsyth Institute (Cambridge, MA). Assays were carried out on a Luminex 100 Bio-Plex Platform. Immediately prior to the initiation of study measurements the Bio-Plex platform underwent a complete on-site maintenance cycle and operational qualification by Luminex field engineers. Daily and weekly performance qualification was continuously verified by Forsyth Institute technicians during the seven week analytical period.

Assay kits provided by the commercial vendors consisted of 5 panels: 1) “**10-plex**” **Panel** of pro- and anti-inflammatory cytokines (TNF-α, IL-1β, IL-6, IFN-γ, IL-4, IL-10, IL-2, IL-5, IL-8, GM-CSF) from Invitrogen (Ultrasensitive kit, Invitrogen, Carlsbad, CA); 2) **Matrix Metalloproteinase Panel** (MMP-2, MMP-8, MMP-9) from R&D Systems (R&D Systems, Minneapolis, MN); 3) **Bone Panel** of bone metabolism markers (osteoprotegrin (OPG), leptin, parathyroid hormone (PTH) and insulin) from Millipore (EMD Millipore, Billerica, MA); 4) **Obesity Panel** (adiponectin and C-reactive protein (CRP)) from R&D Systems; and, 5) “**4-plex**” **Panel** (VEGF, IL-17, TNF-α and MCP-1) from R&D Systems.

Single lot numbers of each kit were purchased in bulk in order to minimize analytical variability. Reagents provided in these kits included beads, monoclonal antibodies, standards, assay diluents, biotin-conjugated secondary antibodies, biotin diluent, streptavidin conjugated to the fluorescent protein, R-phycoerythrin (streptavidin-RPE), streptavidin-RPE diluent, washing buffer concentrates, and incubation buffer concentrates as well as the 96-well filter plates.

Samples were thawed directly on the day of analysis. Working wash solutions were prepared from concentrates on a daily basis. Protein standards were prepared, within one hour of beginning the assay, by reconstituting the standard in assay diluent and performing serial dilutions according to manufacturer specifications. To prepare beads for the multiplex assays, each analyte bead solution was mixed with wash solution or bead diluents in an aluminum foil-wrapped test tube as the beads are light-sensitive.

Bead solution, incubation buffer, assay diluents, samples, standards and blanks were pipetted in designated wells using negative volume displacement precision pipettes (Rainin Instrument LLS, Woburn, MA). Plates were incubated at 4°C, overnight, on an orbital shaker (IKA Werke, Staufen, Germany) set to 600 rpm in order to keep beads suspended. After washing, diluted biotinylated detector antibody was added into each well, followed by incubation and washing. Streptavidin-PRE solution was added into each well after washing; the instrument was calibrated, a standard curve was created, and the observed concentrations of samples were calculated.

### Data Analysis

Statistical analyses were performed to summarize data descriptively and included means, standard deviations and percent coefficient of variation (%CV; relative standard deviation). Pearson correlations between serum and saliva measures were performed on log-transformed data so as to approximately normalize the population frequency distributions of the measurements. In our initial processing of these data we did consider other measures of association. As our log transformed concentrations are nearly normally distributed, and as we intend to use linear regression (and to adjust for other covariates) in other analyses, we chose to use the Pearson correlation (which has close ties to multivariate normality and linear models) to summarize the association between the serum and saliva concentrations. Substantively similar results were obtained with Kendall’s tau correlation. Statistical analyses were performed using SAS V.9.2 (Carey, NC). Further calculations and statistical procedures are described where relevant below.

### Sample Dilution and Diluents

We determined the single most appropriate minimum required dilution (MRD) for each multiplex panel which would allow a maximum number of samples to generate measurements within the linear calibration range [14]. To determine this, multiple cryogenic straws of QC materials and 20 representative sera and saliva samples were analyzed at multiple serial dilutions. The dilution with the highest percentage of measurements falling within the dynamic range was selected. During initial method validation it became apparent that observed recoveries were poor for several analyte panels and alternative sample diluents containing additives were tested to improve recovery and reduce sample matrix effects in both serum and saliva. These additives were prepared into assay sample diluents and included 5–25 mM EDTA, 0.05% Tween 20, 0.1 mM nitric acid and 0.1 mg/ml proteinase K which had been previously suggested to improve uniplexed protein assays [15]–[17]. Pretreatments were evaluated by performing recovery and LLOQ experiments (as described below) on samples with and without alternative diluents/pretreatments. Dilution factors and diluents were selected for each panel based on maximizing recovery of the analytes.

### Recovery

We performed recovery studies by standard additions methodology [18]. For each multiplex panel the highest level assay calibrator was spiked into authentic serum and saliva samples at a ratio of 1∶20. A corresponding baseline sample was spiked with blank matrix material consisting of 3 g/dL bovine serum albumin (BSA) in isotonic saline (for serum recovery experiments) or 1 mg/mL BSA in isotonic saline (for saliva recovery experiments). Six replicates of each baseline and spiked sample were analyzed and the % recovery was calculated as:
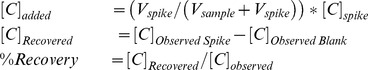
where [*C*] is concentration, V is volume, ‘Observed’ is the measured concentration and ‘spike’ is the highest level assay calibrator.

### Lower Limit of Quantification (LLOQ)

During initial method validation it was apparent that normal serum and saliva levels were near or below the lower limit of quantification (LLOQ) for some analytes. To establish the LLOQ for each analyte, empirical LLOQ determinations were performed as we have described previously in other studies [19]. Briefly, samples containing known levels of analyte for each panel were generated by spiking small volumes of assay calibrators into authentic serum and saliva as described for recovery studies. Decreasing concentrations of analytes were achieved by performing serial dilution using 3 g/dL BSA for serum and 1 mg/mL BSA for saliva. Each experiment consisted of 54 individual samples; 6 replicates of blank matrix, 6 replicates of baseline sample (unspiked serum or saliva sample) and 6 replicates each of 7 serial dilutions (1∶1 to 1∶64) of the spiked sample. For each analyte, the concentration of the 1∶64 dilution was below the lowest calibrator level of the assay. Replicate samples of each dilution were independently pretreated/diluted in the appropriate diluents prior to multiplex analysis.

For each analyte, the known concentrations (and dilutions thereof) were plotted on the x-axis and compared with the %CV of six replicates on the y-axis using SigmaPlot ver. 9.01. The nonlinear trendline was plotted and fitted using a 3-parameter, exponential decay model (*y = y_0_ + ae^−bx^*) and a nonlinear least squares approach to optimize goodness of fit. This nonlinear regression gave the highest correlation of the available SigmaPlot models. The LLOQ was interpolated for each analyte as the concentration in which the %CV equaled 20%. If the 20% threshold was not breached, we accepted the manufacturers reported LLOQ.

### Method Performance

Each QC sample was assayed in duplicate on each multiplex plate and for each of the 5 panels. Daily batches consisted of 2 plates of serum samples and two plates of corresponding saliva samples measured over 7 consecutive weeks. Average within-run CV was calculated from at least 4 replicates of each QC specimen per batch. Unadjusted between-run CV was calculated from at least 28 replicates across all 7 batches.

Intra-assay (within-run, plate-specific) and inter-assay (between-run, plate-to-plate) imprecision as well as trending of the data was evaluated across the study using QC specimens which were analyzed in multiple replicates within each plate. We calculated plate-specific means and %CVs for the sample cohort and QC measurements as well as means and CVs for all plates within the sample set. To characterize the variability of sample levels relative to the imprecision of QC samples we calculated the ratio of analytical-to-inter-individual variability (A/I) defined as the %CV of QC measurements divided by the %CV of all sample measurements.

There was significant plate-to-plate variation in the mean analyte concentrations of participant samples and QC materials. Quantitative and categorical demographic variables were tested across plates by ANOVA F-test and the chi-square test of independence, respectively. As a means of filtering batch-to-batch imprecision the conversion from fluorescent intensity (FI) values to observed concentration (OC) values was followed by an adjustment process at the plate level using the QC replicates. Adjustments were separate for each analyte. After adjustment, the plate means of the log OC of the QC replicates are equal. Briefly, the batch adjustment procedure for each plate and each analyte was to (1) convert all OC to log scale; (2) compute grand mean and plate means of QC replicates; (3) compute residuals (plate means - grand mean); (4) subtract residuals from each log OC to produce `adjusted log OC’; and, (5) exponentiate to produce an `adjusted between Run CV’.

Following this batch adjustment we examined each plate by traditional QC algorithms. We generated Levey-Jennings type plate-to-plate plots of QC values across 7 batches of 4 plates per day (two containing serum samples and two containing homologous saliva samples), constituting 28 total plates. Run acceptability was based upon conventional Westgard rule interpretation [20]–[22], and the samples from plates that ‘failed’ Westgard rule interpretation were excluded from further analyses.

## Results

### Dilution, Recovery and LLOQ

**Table 1** explains the minimum required dilution (MRD) and diluents employed for each panel. The Bone and 4-PLEX panels were found to require no dilution while the MMP panel in both serum and saliva required a 1∶10 dilution to bring analyte levels into the calibration range of the assay. The obesity panel in serum required a 1∶500 dilution to bring CRP and adiponectin levels into the calibration range of the assay while no dilution was required for saliva samples.

**Table 1:** Dilution factors and diluents for each multiplexed assay panel.

		Serum		Saliva
Panels	Dilution	Diluent	Dilution	Diluent
MMP	1∶10	1 mM NA in 0.05% Tween in assay diluent	1∶10	assay diluent
Bone	None	None	None	None
hs10-Plex	1∶2	25 mM EDTA in 0.05% Tween in assay diluent	1∶2	assay diluent
Obesity	1∶500	assay diluent	None	None
2-Plex	None	25 mM EDTA in 0.05% Tween in assay diluent	None	assay diluent

MMP Panel: MMP-2, MMP-8 and MMP-9; Bone Panel: OPG, Leptin, PTH and Insulin; 10-Plex: IL-1β, IL-10,

IL-6, GM-CSF, IL-5, IFN-γ, TNF-α, IL-2, IL-4 and IL-8; Obesity Panel: Adiponectin and CRP; 2-Plex Panel: TNF-α and MCP-1.

**Figure 1:**
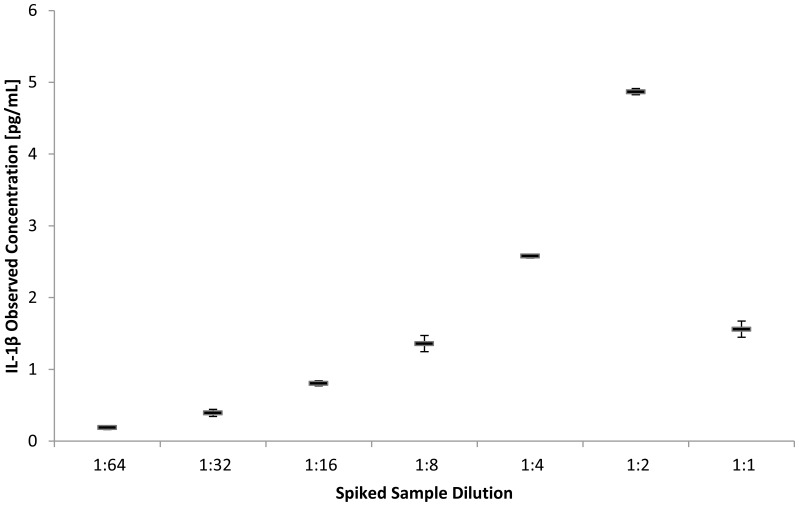
Observed concentration of IL-1β in serum sample spiked 1∶20 with highest calibrator and then diluted and analyzed in replicate. Initial 1∶2 dilution causes a 3 fold increase in observed concentration followed by 50% decreases with each successive serial dilution.

For the 10-plex panel in serum the first two-fold dilution (1∶2) of samples was observed to increase the measured concentration of IL-1β, IL-6, IL-10, IFN-γ, GM-CSF and IL-8 by a factor of 2–4 while further two-fold serial dilutions (1∶4 to 1∶64) resulted in decreasing concentrations. **Figure 1**, by example, illustrates the effect of serial dilution on the observed concentration of IL-1β in serum. We interpreted these results, and similar results in saliva, to indicate that some level of matrix interference effect existed for the 10-plex panel and we therefore assessed assay recovery for all analytes, in all panels, by standard additions methodology. Recovery estimates on undiluted serum demonstrated that all the 10-plex cytokines had recoveries of <34% as shown in **Table 2**. Following 1∶2 dilution using kit sample diluents augmented with different additives, most 10-plex analytes demonstrated a 2–50 fold increase in recovery. The greatest increase in recovery was obtained using 25 mM EDTA and 0.05% Tween-20 in assay sample diluent and this diluent/additive was used for all successive samples including calibrators. TNF-α was the only analyte that failed to show an increase in recovery upon dilution/pretreatment (15% in neat serum versus 19% in 1∶2 diluted/pretreated serum). Based upon this poor recovery we initiated the use of the 4-plex panel specifically to improve measurement of TNF-α. This alternative panel demonstrated 100% recovery of TNF-α in serum and 93% recovery in saliva (**Table 3**). The performance parameters for TNF-α on both the 10-plex and the 4-plex are henceforth reported. Only TNF-α and MCP-1 are reported from the 4-plex panel as we did not obtain valid performance data for IL-17 or VEGF using this panel.

**Table 2:** Comparison of percent recoveries of 10plex analytes in undiluted (neat) serum and following two-fold dilution in sample diluent.

10-PlexAnalyte	Neat Serum	25 mM EDTA/0.05% Tween-20(1∶2 dilution)
IL-1β	12%	46%
IL-10	2%	117%
IL-6	16%	94%
GM-CSF	17%	72%
IL-5	22%	168%
IFNG-γ	34%	51%
TNF-α	15%	19%
IL-2	30%	63%
IL-4	22%	54%
IL-8	34%	107%

EDTA, ethylenediaminetetraacetic acid disodium salt.

**Table 3:** Multiplex assay performance characteristics including the kit manufacturer’s calibration range, and stated lower limit of quantification (LLOQ), empirically derived LLOQ and percent recovery in serum and saliva.

Panel	Analyte	CalibrationRange	Unit	KitLLOQ	EmpiricalLLOQ	Serum %Recovery	Saliva % Recovery
Bone	Insulin	0.08–250	ng/mL	0.05	*	125	184
	Leptin	0.016–300	ng/mL	0.12	*	7	59
	OPG	0.50–8500	pg/mL	1.42	*	156	36
	PTH	0.55–9800	pg/mL	0.3	*	88	38
MMP	MMP-1	9.13–6800	pg/mL	6.3	37	58	42
	MMP-2	9.7–55,000	pg/mL	7.5	400	ND	102
	MMP-8	12.5–80,000	pg/mL	5.0	*	20	ND
	MMP-9	8.1–47,900	pg/mL	11.0	*	107	ND
Obesity	Adiponectin	0.37–270	ng/mL	0.0198	*	ND	92
	CRP	0.03–21	ng/mL	0.0019	*	ND	56
10 Plex	GM-CSF	0.20–387	pg/mL	<1.0	1.1	72	56
	IFNγ	0.10–145	pg/mL	<1.0	*	51	168
	IL-10	0.20–485	pg/mL	<1.0	*	117	8
	IL-1β	0.10–211	pg/mL	<1.0	*	46	85
	IL-2	0.10–273	pg/mL	<1.0	*	63	128
	IL-4	0.20–417	pg/mL	<1.0	*	54	112
	IL-5	0.20–393	pg/mL	<1.0	*	168	70
	IL-6	0.10–133	pg/mL	<1.0	*	94	116
	IL-8	0.20–356	pg/mL	<1.0	*	107	ND
	TNFα	0.10–212	pg/mL	<1.0	*	19	83
4-Plex	MCP-1	6.2–2100	pg/mL	0.5	*	ND	ND
	TNFα	2.3–4500	pg/mL	0.3	*	100.4	93.24

ND, not determined as baseline sample level was>highest calibrator.

* all replicate samples achieved CV <20% and therefore the manufacturer’s stated LLOQ is accepted.

In saliva 10-plex panels, two-fold dilution generated similar increases in measured analyte concentration and % recovery; however, inclusion of additives in the assay sample diluents did not further improve recovery and were therefore not used (data not shown). For the remaining panels, dilution/additive used (**Table 1**) resulted in only modest increases in recovery for MMPs (1 mM nitric acid/0.05% Tween-20) and 4-plex panels (25 mM EDTA and 0.05% Tween-20); however, these improvements were typically less than 25%.

**Figure 2:**
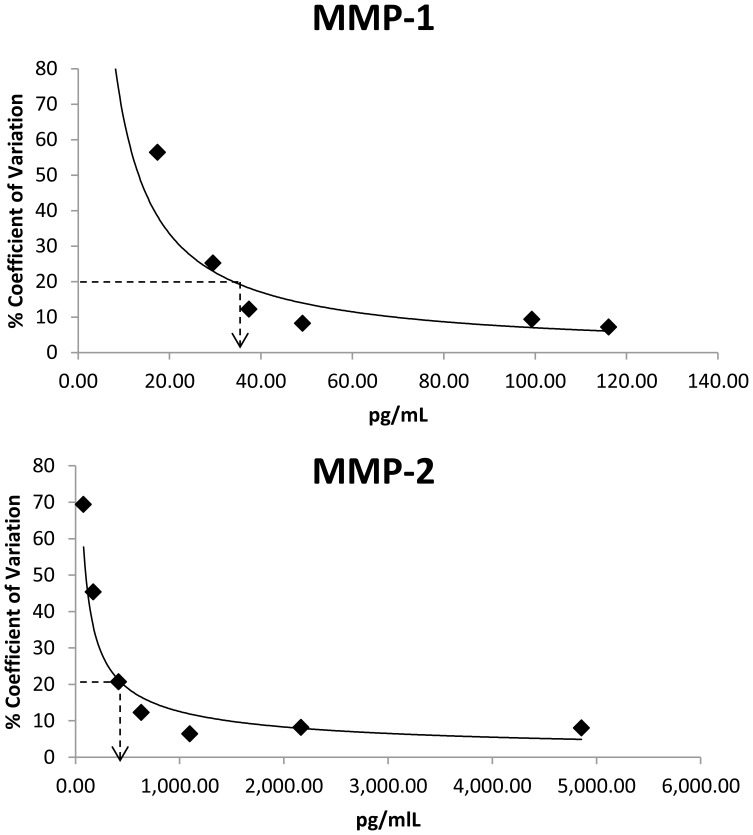
Empirical estimation of the lower limit of quantification (LLOQ) for matrix metalloproteinase 1 (MMP-1) and MMP-2. The x-axis is analyte concentration and the y-axis is the coefficient of variation of six replicate measurements of authentic serum spiked with analyte by standard additions methodology.

**Table 3** provides the multiplex assay performance characteristics including the kit manufacturer’s calibration range, manufacturer’s stated LLOQ, empirically determined LLOQ and the percent recovery obtained in serum and saliva according to final sample dilution and assay conditions described in Table 1. In the empirical LLOQ estimates, 17 of 20 serum analytes demonstrated a %CV <20 at all analyte levels tested and we therefore accepted the manufacturer’s stated LLOQ. GM-CSF had an empirical LLOQ of 1.1 pg/mL, close to the manufacturers stated limit of <1.0 pg/mL. Only MMP-1 (37 pg/mL) and MMP-2 (400 pg/mL) had empirical LLOQ estimates greater than the manufacturer’s stated LLOQ. The empirical LLOQ plots for serum MMP-1 and MMP-2 are shown in **Figure 2**. For serum MMP-2, adiponectin, CRP and MCP-1, and for salivary MMP-8, MMP-9, IL-8 and MCP-1, cohort samples were found to have analyte levels that exceeded the upper limit (highest calibrator) of their calibration curves. Hence, the highest assay calibrator was insufficient to augment the analyte concentration by spiking and recovery estimates in neat samples were not possible.

**Table 4** provides estimates of imprecision generated from pooled human serum and saliva QC materials. The average within-run %CVs ranged from 5.8% (Insulin) to 41.05% (IL-1β) while between-run imprecision ranged from 29.3% (IL-10) to 103.5% (IFN-γ). Upon encountering significant plate-to-plate variations in the means of samples and QC materials, we examined whether demographic variables were randomly distributed across the plates and no significant differences were found. Based on these imprecision estimates, several plates were rejected by conventional algorithms for interpreting quality control measures. As plate specific medians of QC samples and cohort serum samples were significantly correlated, an ‘adjusted between-run CV’ was estimated as described in the methods section. The batch-adjusted serum CV estimates were used to rank each analyte from lowest to highest imprecision within Table 4. Overall, we targeted limits of 20% CV as acceptable, using 25% CV at LLOQ to define acceptability as suggested by Findlay et al. [14], [23]. Based on these limits, 18 of 22 serum analytes were deemed acceptable. Serum CRP (20.65% CV) exceeded the 20% threshold; however, the mean level of CRP in our QC material was 1.3 pg/mL as measured before correction for 1∶500 MRD. This level was in the lower 5% of the calibration curve (0.3 to 21.0 ng/mL) and considered to be at or near the LLOQ. Therefore, <25% CV was deemed acceptable performance for this analyte. In saliva, 9 of 22 analytes had CVs <20% and were deemed acceptable. The CV of saliva leptin was <25% CV however its mean level was well above the LLOQ and it was therefore not considered acceptable.

**Table 4:** Estimates of imprecision of multiplexed analytes in serum and saliva using pooled quality control materials.

			Serum					Saliva			
Panel	Analyte	N	Average Within run CV	Unadjusted Between Run CV	Adjusted Between Run CV	AI	N	Average Within run CV	Unadjusted Between Run CV	Adjusted Between Run CV	AI
Bone	Insulin	32	5.80	30.19	**6.23**	0.06	31	20.33	38.20	**16.59**	0.13
MMP	MMP_8	32	7.17	31.66	**7.10**	0.07	31	10.91	25.55	**11.58**	0.14
MMP	MMP-2	32	8.69	25.26	**7.22**	0.26	20	17.79	48.73	**16.50**	0.16
MMP	MMP-9	30	7.24	25.33	**7.27**	0.13	31	8.29	14.19	**8.67**	0.16
4Plex	TNF-α	28	10.62	84.78	**9.24**	0.21	29	14.57	88.61	**14.74**	0.11
Bone	Leptin	32	7.79	26.32	**9.80**	0.10	31	29.38	43.00	24.42	0.39
Bone	PTH	30	10.13	36.40	**11.30**	0.08	29	27.78	54.29	29.46	0.24
4Plex	MCP-1	30	4.36	23.69	**12.00**	0.26	29	13.39	18.61	**12.91**	0.13
Bone	OPG	30	10.01	35.10	**12.06**	0.29	31	16.21	56.52	**15.18**	0.15
10Plex	TNF-α	30	14.88	53.32	**14.12**	0.04	30	46.54	55.54	73.52	0.41
10Plex	IL-10	32	16.52	29.31	**14.42**	0.03	30	39.54	51.03	109.38	0.20
10Plex	IL-4	30	14.85	72.03	**15.13**	0.04	30	40.05	52.20	98.64	0.23
10Plex	IL-6	30	16.46	46.54	**16.44**	0.03	30	38.98	58.39	55.49	0.19
10Plex	Adiponectin	32	21.30	34.38	**17.07**	0.30	26	8.13	40.41	**5.90**	0.04
10Plex	IL-2	30	20.05	36.13	**18.03**	0.04	30	38.51	45.11	107.35	0.23
10Plex	IL-5	30	20.80	31.10	**19.33**	0.03	30	47.91	58.15	119.11	0.22
10Plex	IL-8	32	22.68	38.66	**19.72**	0.15	–	–	–	–	–
Obesity	CRP	30	25.32	36.02	**20.65**	0.25	24	23.10	115.51	**15.37**	0.09
10Plex	IFN-γ	30	24.58	103.45	25.22	0.05	30	42.47	72.56	94.13	0.13
10Plex	GM-CSF	30	30.28	64.34	31.57	0.08	30	42.14	49.52	102.09	0.22
10Plex	IL-1β	29	41.05	89.40	48.58	0.25	–	–	–	–	–

CV, % coefficient of variation; AI, ratio of analytical imprecision (CV of QC materials) over interindividual variation (CV of all participant values) with a target value of <0.25; QC data for IL-1β and IL-8 were insufficient in saliva, no data is reported; Bolded Adjusted Between Run CV indicate analytes that are in the acceptable range (<25%) for imprecision.

The 10-plex analytes in our serum QC materials were repeatedly measured below the manufacturer’s LLOQ (<1.0 pg/mL) yet still above the lowest calibrator. The imprecision estimates described above were therefore generated at the extreme low range of the assay and should be interpreted carefully because the high CVs demonstrate the expected loss of precision when quantifying samples at the extreme ends of the assay’s range [24]. In saliva, all the QC material 10-plex analytes were similarly measured at or near the LLOQ with the exception of IL-8 and IL-1β which were measured repeatedly above the highest calibration point of the assay (>273 and 356 pg/mL respectively, see table 3). Given the calibration curves were sigmoidal and plateaued at the upper extreme, we chose not to extrapolate and imprecision estimates were not calculated.

The ratio of analytical-to-inter-individual variability (A/I ratio) calculated for each analyte provides a point of comparison for the amount of analytical imprecision relative to the inter-individual variation for each analyte. Studies of biological variability considered minimal analytical imprecision to be less than half intraindividual variation and require that intraindividual variation be less than half interindividual variation in order for a biomarker to be minimally useful in distinguishing longitudinal differences within a person or distinguishing person-to-person differences within a population [25], [26]. Lacking sufficient intraindividual replicates, we nominally considered a value of 0.25 (i.e., ½×½) to be of minimal acceptability for this parameter. Of the 17 serum analytes with acceptable imprecision MMP-2, MCP-1, OPG and adiponectin had an A/I ratio greater than 0.25 indicating that the analytical imprecision is very high relative to interindividual differences and these specific assays may be of limited value in examining biomarker differences between individuals.

**Tables 5** **–** **8** describe the numbers of serum and saliva samples tested in the study and the number of usable analyte measurements after removal of samples lost to failed QC and samples which had levels outside the quantifiable range of the assay. For serum samples (**Tables 5** **–** **6**), a total of 1320 samples (baseline and follow-up, inclusive) were sent for testing and the percentage of quantifiable results ranged from 32% (n = 433 for IL-5) to 100% (n = 1320 for IL-8). Losses to failed QC resulted from single plates being rejected by Westgard QC algorithm rules wherein rule violations included one QC replicate >3 SD away from the sample mean (1–3 s fail rule) or two QC replicates >2 SD away from the sample mean (2–2 s fail rule). In serum, out-of-range values resulted almost exclusively from sample concentrations below the LLOQ with only CRP generating substantial numbers of measurements (13.7%) above the linear range.

**Table 5:** Descriptive statistics of all study participant serum samples tested for Bone Panel (osteoprotegrin (OPG), leptin, parathyroid hormone (PTH) and insulin), Matrix Metalloproteinase Panel (MMP-2, MMP-8, MMP-9) and Obesity Panel (adiponectin and C-reactive protein (CRP)).

Analyte	Insulin	Leptin	OPG	PTH	MMP-2	MMP-8	MMP-9	Adiponectin	CRP
(Unit)	(ng/mL)	(ng/mL)	(pg/mL)	(pg/mL)	(pg/mL)	(pg/mL)	(pg/mL)	(ng/mL)	(ng/mL)
Total N Tested	1320	1320	1320	1320	1320	1320	1320	1320	1320
Total N Pass QC	1320	1320	1226	1226	1320	1320	1226	1320	1226
% below	2.5	0.61	0.08	0	1.59	1.89	0.49		0.24
% above	0	0	0	0	0.61	0	1.88	0.83	13.7
N	1287	1312	1225	1226	1290	1295	1196	1307	1055
Mean(SD)	0.191(0.211)	4.24(4.02)	334.9(140.5)	32.14(43.69)	192,490(47,910)	7,910(7,776)	146,937(83,364)	19,829(11,440)	2,908(2,362)
Min	0.054	0.16	7.38	0.903	3,618	637.2	381.5	42.39	12.94
25^th^ percentile	0.109	1.63	246.1	21.72	163,222	3,440	85,674	12,123	1,056
Median	0.14	3.16	301.1	27.92	194,419	5,690	129,839	16,984	2,238
75^th^ percentile	0.196	5.57	396.1	35.56	222,013	9,217	188,153	24,690	4,217
Max	3.84	50.77	1,369	1,243	386,209	113,240	533,844	111,687	12,847

Total N Tested; number of participant samples sent for testing, Total N pass QC; number of participant samples after removal of failed QC batches,

% above/below; percent of samples to pass QC but fall above or below the quantifiable range of the assay based on table 3.

**Table 6:** Descriptive statistics of all study participant serum samples tested for “10-plex” Panel (TNF-α, IL-1β, IL-6, IFN-γ, IL-4, IL-10, IL-2, IL-5, IL-8, GM-CSF) and “4-plex” Panel (TNF-α and MCP-1).

Analyte	GM-CSF	IFN-γ	IL-10	IL-1β	IL-2	IL-4	IL-5	IL-6	IL-8	TNF-α^1^	MCP-1	TNF-α^2^
(Unit)	(pg/mL)	(pg/mL)	(pg/mL)	(pg/mL)	(pg/mL)	(pg/mL)	(pg/mL)	(pg/mL)	(pg/mL)	(pg/mL)	(pg/mL)	(pg/mL)
Total N Tested	1320	1320	1320	1320	1320	1320	1320	1320	1320	1320	1320	1320
Total N Pass QC	1226	1226	1320	1109	1226	1226	1226	1226	1320	1226	1226	1138
% below	60.44	33.77	17.05	33.27	4.32		64.68	4.08	0	0	0	3.16
% above	0.73	0	0	0	0	0.08	0	0.24	0	0.16	0	0
N	476	812	1095	740	1173	1225	433	1173	1320	1224	1226	1102
Mean(SD)	33.34(80.01)	0.746(3.34)	10.89(45.84)	1.32(2.35)	2.79(11.94)	5.73(21.39)	2.70(10.64)	4.33(20.31)	13.12(17.57)	4.16(14.83)	143.3(66.49)	1.72(0.758)
Min	1.46	0.111	0.276	0.083	0.164	0.39	0.254	0.162	0.592	0.181	1.24	0.56
25^th^ percentile	3.46	0.223	0.598	0.37	0.44	1.26	0.454	0.515	6.51	0.935	99.34	1.27
Median	7.23	0.316	1.01	0.622	0.681	1.68	0.657	0.877	9.28	1.43	134.5	1.59
75^th^ percentile	21.1	0.486	2.7	1.25	1.39	2.82	1.28	1.89	13.82	2.64	179.1	1.98
Max	888	86.5	633.5	28.45	241.4	360.2	129.5	379	322.6	350.3	471.6	13.14

Total N Tested; number of participant samples sent for testing, Total N pass QC; number of participant samples after removal of failed QC batches,

% above/below; percent of samples to pass QC but fall above or below the quantifiable range of the assay based on table 3.

1– TNF-α assayed as part of the 10-Plex.

2– TNF-α assayed with MCP-1.

**Table 7:** Descriptive statistics of all study participant saliva samples tested for Bone Panel (osteoprotegrin (OPG), leptin, parathyroid hormone (PTH) and insulin), Matrix Metalloproteinase Panel (MMP-2, MMP-8, MMP-9) and Obesity Panel (adiponectin and C-reactive protein (CRP)).

Analyte	Insulin	Leptin	OPG	PTH	MMP-2	MMP-8	MMP-9	Adiponectin	CRP
(Unit)	(ng/mL)	(ng/mL)	(pg/mL)	(pg/mL)	(pg/mL)	(pg/mL)	(pg/mL)	(ng/mL)	(ng/mL)
Total N Tested	1133	1133	1133	1133	1133	1133	1133	1133	1133
Total N Pass QC	1133	1133	1133	1059	820	1133	1133	1062	968
% below	5.83	9.89	0.35	2.83	83.29	0.44	0.09	0.47	2.58
% above	0	0	0	0	0	6.47	31.71	1.6	0.1
N	1067	1021	1129	1029	136	1050	768	1040	942
Mean(SD)	0.484(0.579)	0.362(0.205)	167.6169.9)	21.425.7)	5,6975,840)	214,728179,177)	224,126122,263)	22.8132.3)	0.9921.66)
Min	0.037	0.06	2.84	0.449	2,134	1,259	254.9	0.068	0.002
25^th^ percentile	0.146	0.208	72.43	4.87	2,840	79,866	125,687	6.13	0.154
Median	0.278	0.321	115.5	11.2	3,487	161,302	214,383	11.8	0.438
75^th^ percentile	0.622	0.467	203.7	27.8	6,015	296,368	317,275	25.2	1.12
Max	5.31	1.57	2,293	185.6	33,807	1,049,129	532,426	276.7	25.9

Total N Tested; number of participant samples sent for testing, Total N pass QC; number of participant samples after removal of failed QC batches

% above/below; percent of samples to pass QC but fall above or below the quantifiable range of the assay based on table 3.

**Table 8:** Descriptive statistics of all study participant saliva samples tested for “10-plex” Panel (TNF-α, IL-1β, IL-6, IFN-γ, IL-4, IL-10, IL-2, IL-5, IL-8, GM-CSF) and “4-plex” Panel (TNF-α and MCP-1).

Analyte	GM-CSF	IFN-γ	IL-10	IL-1β	IL-2	IL-4	IL-5	IL-6	IL-8	TNF-a^1^	MCP-1	TNF-a^2^
(Unit)	(pg/mL)	(pg/mL)	(pg/mL)	(pg/mL)	(pg/mL)	(pg/mL)	(pg/mL)	(pg/mL)	(pg/mL)	(pg/mL)	(pg/mL)	(pg/mL)
Total N Tested	1133	1133	1133	1133	1133	1133	1133	1133	1133	1133	1133	1133
Total N Pass QC	1133	1133	1133	1133*	1133	1133	1133	1133	1133*	1133	1039	1042
% below	74.03	37.99	32.36	0.53	5.21	2.3	68.55	0.71	0	3.18	0.29	5.37
% above	0.88	1.5	0.18	11.58	0.53	0.97	0.09	1.86	81.79	2.74	3.37	0
N	284	685	763	994	1067	1094	355	1102	206	1065	1001	986
Mean(SD)	39.77(100.2)	21.48(134.3)	11.8(55.0)	61.3(69.5)	9.64(44.8)	26.0(111.3)	17.1(60.9)	14.2(40.7)	413.2(188.5)	20.6(36.8)	374.1(360.3)	5.27(7.17)
Min	1	0.073	0.178	0.48	0.119	0.35	0.178	0.106	0.74	0.132	1.77	0.526
25^th^ percentile	3.61	0.213	0.706	21.1	0.711	2.25	0.682	1.59	254	4.3	143.6	1.97
Median	8.15	0.836	1.62	38.1	1.32	4.98	1.84	4.14	436.1	8.96	244.7	3.32
75^th^ percentile	27.04	4.61	3.93	73.0	3.45	11.63	5.05	10.46	578.2	19.01	471.3	5.83
Max	799.5	2,425	669.1	421.6	872.1	1,854	480.4	621.6	705.4	411.1	2,303	86.88

Total N Tested; number of participant samples sent for testing, Total N pass QC; number of participant samples after removal of failed QC batches.

% above/below; percent of samples to pass QC but fall above or below the quantifiable range of the assay based on table 3.

1– TNF-α assayed as part of the 10-Plex.

2– TNF-α assayed with MCP-1.

* - the QC material was insufficient for evaluation of these analytes, all data within analytic range is reported and has not been adjusted by batch.

For saliva (**Tables 7** **–** **8** **)**, of the 1,133 samples (baseline and follow-up, inclusive) tested, the percentage of quantifiable results ranged from 0.2% (n = 34 for IL-8) to 99% (n = 1,129 for OPG). As discussed above, IL-8 and IL-1β analyte levels in our QC specimens were above the highest calibrator, QC measurements could not be evaluated, batch correction was not performed and the number of “usable” measurements is calculated from the unadjusted data. For IL-8 and IL-1β, 81% and 12%, respectively, of all saliva sample measurements were above the linear range respectively. For IL-8 this resulted in only 206 detectable measurements available. For the remaining saliva analytes, substantial numbers of out-of-range values were found both below and above the linear range.

**Table 9** describes the relationships between serum and saliva analyte levels. The Pearson Product-Moment correlation coefficient (r) gives a crude estimation of the association between serum and salivary analyte concentrations. The strongest correlation is seen for CRP (r = 0.66). Insulin and adiponectin show weaker correlations of r = 0.29 and r = 0.31, respectively. There is a weak correlation for OPG (r = 0.12). The saliva to serum ratio indicates the relative analyte concentrations between fluid compartments. Saliva:serum ratios are less than one for leptin, OPG, PTH, MMP-2, adiponectin, CRP and GM-CSF, suggesting that these analytes are present in saliva in much lower concentrations than in serum. In contrast, insulin and almost all the cytokines are significantly higher in salivary samples (saliva:serum ratio>1). MMP-8, IL-8 and IL-1β, in particular, are much higher in saliva and have ratios that exceed 30∶1.

**Table 9:** Saliva:Serum Ratio and Pearson Correlation for all samples.

Panel	Analyte	N	Medianof Ratios	Pearsoncorrelationr	LCL	UCL
Bone	Insulin	1047	1.82	0.29	0.23	0.34
	Leptin	1015	0.10	0.02	–0.04	0.08
	OPG	1054	0.38	0.12	0.06	0.18
	PTH	955	0.40	0.01	–0.05	0.08
MMP	MMP-2	134	0.02	0.25	0.08	0.40
	MMP-8	1035	29.62	0.06	7.0E−04	0.12
	MMP-9	696	1.69	–0.03	–0.11	0.04
Obesity	Adiponectin	1030	6.7E−04	0.31	0.25	0.36
	CRP	781	1.5E−04	0.66	0.53	0.62
10-plex	GM-CSF	113	0.97	0.27	0.08	0.43
	IFN-γ	416	2.64	–0.01	–0.11	0.09
	Il-10	639	1.35	0.06	–0.01	0.14
	Il-1β*	559	62.57	–0.03	–0.23	0.17
	Il-2	957	1.78	0.07	3.1E−03	0.13
	Il-4	1025	2.57	–0.04	–0.10	0.03
	Il-5	113	3.07	0.20	0.01	0.37
	Il-6	979	4.22	0.05	–0.01	0.11
	Il-8*	206	46.17	–0.33	–0.51	–0.10
	TNF-α	997	5.29	8.0E−03	–0.05	0.07
4-plex	MCP-1	919	1.89	0.03	–0.04	0.09
	TNF-α	887	2.17	0.02	–0.05	0.08

All values are batch adjusted.

Median of Ratios – median of ratios computed for each participant.

* Saliva values for these analytes are not adjusted due to insufficient QC data.

Pearson Correlation of natural log adjusted values, LCL, UCL lower and upper 95% confidence interval.

## Discussion

Our goal in this study was to use multiplex technology to simultaneously measure a relatively large set of protein biomarkers in serum and homologous saliva. We developed sample dilution and pre-treatments that improved recovery estimates for many analytes. We confirmed the lower limit of quantification for each analyte. We determined that 17 of 20 biomarkers in serum and 9 of 20 biomarkers in saliva demonstrated acceptable precision. We examined a large cohort of well defined specimens and determined the percentage of cohort samples having levels within the reportable range. Finally, we determined the ratio of levels in saliva to serum, and assessed correlations between saliva and serum.

Before initiating analysis of participant samples, we attempted to characterize the performance characteristics of these methods, guided by the kit manufacturer’s protocols. These initial efforts indicated poor performance in many of the assays we evaluated. We therefore undertook efforts to optimize these assays. In order to bring endogenous analyte levels into the analytical range of the assays, we established minimum required dilutions (MRD) ranged from 1∶1 (no dilution) to 1∶500 in serum while saliva MRDs ranged from 1∶1 to 1∶10. Upon dilution we identified significant matrix effects for many analytes and therefore, different pre-treatment diluents were selected to minimized these effects and improve the recovery of the analytes. Matrix effects are interferences in the measurement of a target analyte caused by non-analyte components of complex milieus such as serum and saliva. Matrix effects are an especially critical issue when multiplexing analytes since interfering substances such as non-specific sample proteins may affect high abundance targets differently than targets with lower concentrations. While the paradigm dictates that matrix effects may be more of a problem in serum with its high protein concentration, our results suggest that saliva poses the same limitation. Indeed, salivary components are well known to interact with other components to form so called “heterotypic complexes” [27]. Such complex formation could obscure epitopes and reduce detection of analytes.

We have shown here that careful determination of MRDs and pretreatment/dilution with various diluents can reduce matrix effects and increase the recoveries as shown in Table 2. We note however that extensive dilution can result in dilution of low abundance analytes to below detection limits. Furthermore, we note that MRDs and pretreatments are matrix specific in that pretreatments which are effective in serum are not necessarily effective in saliva. Finally, even after such extensive testing for the optimal conditions for processing the targeted analytes in this study, assay conditions for some of the molecules were still not satisfactory, which suggests that other factors including the microsphere antibodies and their sensitivity and specificity should also be considered.

We evaluated imprecision of study materials using coefficients of variation (CVs) as a measure of variability. Most analytes had acceptable levels of within-run imprecision for QC materials (within plate %CV <20%); however, between-run (plate-to-plate) imprecision was >20% for all analytes. We tested whether this variation could have been due to differences in demographic qualities across plates, and could not identify any factors to explain the differences that were found. Plate specific cohort means co-varied with QC material measurement and conventional QC algorithms rejected more than half of all cohort data. We therefore applied statistical adjustment which considered some of the plate-to-plate variability and increased the number of usable observations. When doing studies with large numbers of samples over time, such batch adjustments may be necessary. After adjustment, traditional application of Westgard rules eliminated only the most errant results. In serum, 17 out of 20 analytes showed acceptable performance defined as adjusted between-run CV of <20% (<25% at LLOQ). MMP-2, MCP-1, OPG and adiponectin each had acceptable imprecision in serum and saliva; however, the ratio of analytical imprecision to interindividual variation indicated that these analytes may be of limited value as biomarkers in a population based or diagnostic setting. Fewer analytes were acceptable in saliva using this method with 9 out of 20 meeting acceptability criteria. Of those saliva analytes rejected, we found that IL-8 and IL-1β were present at significantly higher levels in saliva than in serum. The single MRD therefore yielded insufficient dilution for these analytes and a large proportion of the measurements (IL-8 in particular) were above the linear range of the assay. We suggest that in future assessments of saliva that these cytokines be carefully considered in larger plexed panels where these problems may occur and tested separately so that a more suitable dilution can be achieved without detriment to other 10-plex analytes.

There are many different vendors and platforms now available for multiplex assays. The design, nature of quality control material and imprecision estimates from these sources vary greatly, and reports are almost exclusively about serum or plasma. Hsu et al reported interassay imprecision for a panel of cytokine analytes that ranged from 10.2–19.8% [28]. Liew and colleagues reported interassay imprecision <13% for a panel of protein hormones and cancer antigens; however, it is important to note that all analytes examined were present in the ng/mL to µg/mL range and QC estimates were generated on selected specimens with higher concentrations [29]. Urbanowska et al reported analytical recoveries from 70–130% and interassay imprecision of 10.3 to 29.8%CV on a multiplex cytokine panel using plasma based QC materials that were augmented with recombinant cytokines. Ellington et al used three levels of recombinant QC materials in a study where, although not reporting specific %CV value, >50% of all plates exceeded QC targets ranges [8]. The most detailed examination to date has been performed by Chaturvedi et al who examined 116 inflammation, immune, and metabolic markers across two Luminex bead–based commercial kit manufacturers (Bio-Rad and Millipore) and three specimen types (serum, heparin and EDTA plasma) and found that 19 of 64 Bio-Rad markers and 23 of 90 Millipore markers had CVs for across-batch duplicates greater than 20% on two or more specimen types [10]. Our serum studies agree with these previous reports in that our imprecision estimates indicate that there is significant room for improvement for most analytes. Additionally, our study is one of the first to report performance in saliva, and the results indicate that imprecision may be an even greater challenge in the saliva sample matrix.

In addition to imprecision problems, recovery studies suggest that sample matrix effects can result in significant inaccuracy. This phenomena was also previously observed in a study using a more limited set of analytes and a similarly modified diluent improved the accuracy of the spike recovery for two different multiplex platforms [30]. To more fully quantify accuracy, comparisons should be made to uniplex ELISA assays generally considered as the current gold standard. Many of these comparisons have been conducted and indicate that comparison of randomly selected multiplex assays with ELISA is likely to generate substantial differences in quantitative values [31] due to the use of different capture and reporter antibodies, diluents and serum blockers. Other reports suggest that multiplex assays are further limited in that they only provide accurate quantitation for analytes that are present in relatively large concentrations [9]. Larger scale validation and comparison studies are needed to more comprehensively examine the methodological aspects of these assays in the future.

One of the aims of the larger grant was to compare analyte measures in saliva and serum samples collected at the same time among a defined set of participants in order to determine correlations between biomarker concentrations in saliva and serum. Saliva has been proposed as a convenient medium for monitoring local and systemic inflammatory processes [32]. However, there are a number of known methodological challenges to salivary measurements, and the association of inflammatory marker concentrations between saliva and serum remains unclear. The concentrations of many analytes in saliva can vary depending on the time of day, state of salivary gland simulation, interference from dietary constituents and oral health status [33]–[35]. Our study was able to collect samples from individuals at defined times and under standard conditions to allow these correlations to be assessed. Simple correlation studies have been reported, however further in-depth analyses will be the topic of future papers to assess the utility of salivary samples in characterizing serum levels for the markers we examined. Some markers show promise (e.g., CRP, adiponectin, GM-CSF) and will be explored further in detail in the future. Interestingly, the data also suggests that some of these analytes are in higher abundance in saliva compared to serum. For example, MMP-8 and most of the cytokines are significantly higher in salivary samples. Higher levels of MMP-8 in saliva have been previously reported [36] and are explained by its production by human odontoblasts and dental pulp cells [37]. It is possible that inflammation deriving from new onset or progressing oral or systemic disease could be associated with the presence of high levels of other proteins and/or cytokines in saliva. In some instances, saliva measures may be associated with certain conditions more directly than serum measures. We intend to examine this in subsequent reports. Within the limits of the current work, saliva presents an available and potentially useful biological fluid for monitoring inflammation in humans. The source of biomarkers in saliva needs to be studied in detail since it would be important to understand if these levels simply reflect a less diluted form of the serum content or if there are local mechanisms responsible for the biomarker abundance in saliva.

Strengths of the current study include the large, well-characterized cohort of postmenopausal women. Given their age, these participants provide a great opportunity to study a broad range of biomarkers and will, in further analyses, allow us to explore the biomarkers according to personal characteristics in various levels of health and disease. Importantly, the serum and saliva were obtained from each individual participant at one visit using standardized protocols including careful handling and processing of samples for immediate freezing. A single laboratory completed all bioassays in serum and saliva and attempts to control variation by using the same lot numbers for each biomarker assay kit was another strength. There were a large number of participants assayed and many had measures available at two time points.

There are several limitations of the current study. First, there is a lack of repeat measurements of all samples due to cost. Use of QC replicates allowed us to examine this issue, but further replicates would have been useful. Second, we were not able to perform direct comparison using more traditional assays such as ELISA due to sample limitations and cost. We do have information on some traditional markers (i.e., CRP by nephelometry and insulin by chemiluminescent immunoassay) that will be available for exploration in future analyses. Direct comparison between multiplex and traditional ELISA however is difficult to accomplish. A number of published studies have compared these two methods and it is apparent that certain elements of these assays are pivotal in obtaining similar results from both assays [31], [38]. Factors driving these differences include differences in the clones of monoclonal antibodies used for detection and reporting, differences in surface chemistries (plates, beads, etc.), and variability due to cross-reactivity of antibodies while analyzing multiple ligands simultaneously. Finally, while the focus of this report is on describing the performance of multiplex assay and assay panel kits, results are only relevant in the context of the population, specific disease or condition for which they are measured. The study participants were not selected on any specific condition, they were all community dwelling postmenopausal women who were participants in the WHI Observational Study from the Buffalo NY clinical center. Eligibility for OsteoPerio required presence of at least six teeth and excluded active cancer, certain bone issues within a specified time interval preceding enrollment, and no current use of certain medications known to impact bone (i.e. corticosteroids, amino-bisphosphonates). Some of the analytes tested which had low precision (and low absolute levels) or a poor analytical acceptability as indicated by an AI>0.25, might still have utility in studying conditions or populations where those analytes are present in vastly higher concentration. These findings are only generalizable to similar community dwelling postmenopausal women and further study will be needed to determine if the performance reported here is similar in samples collected from other groups.We have attempted to evaluate, optimize and implement five, bead-based, multiplexed immunoassay panels of 20 protein analytes in serum and homologous saliva. We were able to identify methods to improve performance by sample dilution and pre-treatment additives. Plate-to-plate imprecision was optimized by use of QC material to normalize values and allow use of many plates that may have been rejected by traditional methods. A statistical batch adjustment procedure was developed and applied which filtered much of the batch effect and increased the number of usable measurements. Analysis of the current cohort of samples indicates that several analytes are correlated between serum and saliva and may have utility as potential biomarkers in human health and disease. In order to realize the potential of salivary markers in further human research, there is a need for additional research to further optimize assay performance, develop performance validation parameters and quality control programs, and to standardize assays in various biological fluids of clinical relevance, building on the work we report here.
